# Whole Genome Sequencing of Australian *Candida glabrata* Isolates Reveals Genetic Diversity and Novel Sequence Types

**DOI:** 10.3389/fmicb.2018.02946

**Published:** 2018-12-03

**Authors:** Chayanika Biswas, Vanessa R. Marcelino, Sebastiaan Van Hal, Catriona Halliday, Elena Martinez, Qinning Wang, Sarah Kidd, Karina Kennedy, Deborah Marriott, C. Orla Morrissey, Ian Arthur, Kerry Weeks, Monica A. Slavin, Tania C. Sorrell, Vitali Sintchenko, Wieland Meyer, Sharon C.-A. Chen

**Affiliations:** ^1^Centre for Infectious Diseases and Microbiology-Public Health, Westmead Hospital, Sydney, NSW, Australia; ^2^Westmead Clinical School, Faculty of Medicine and Health, The University of Sydney, Sydney, NSW, Australia; ^3^Centre for Infectious Diseases and Microbiology, Westmead Institute for Medical Research, Westmead, NSW, Australia; ^4^Marie Bashir Institute for Emerging Infectious Diseases and Biosecurity, The University of Sydney, Sydney, NSW, Australia; ^5^Department of Infectious Diseases and Microbiology, New South Wales Health Pathology, Royal Prince Alfred Hospital, Faculty of Medicine and Health, The University of Sydney, Sydney, NSW, Australia; ^6^Centre for Infectious Diseases and Microbiology Laboratory Services, ICPMR, New South Wales Health Pathology, Westmead Hospital, Sydney, NSW, Australia; ^7^National Mycology Reference Centre, SA Pathology, Adelaide, SA, Australia; ^8^Department of Microbiology and Infectious Diseases, Canberra Hospital & Health Services, Australian National University Medical School, Canberra, ACT, Australia; ^9^Department of Microbiology and Infectious Diseases, St Vincent’s Hospital, Sydney, NSW, Australia; ^10^Department of Infectious Diseases, Alfred Health and Monash University, Melbourne, VIC, Australia; ^11^Department of Microbiology, PathWest Laboratory Medicine, Queen Elizabeth II Medical Centre, Perth, WA, Australia; ^12^Department of Microbiology and Infectious Diseases, Royal North Shore Hospital, Sydney, NSW, Australia; ^13^National Centre for Infections in Cancer, Peter MacCallum Cancer Centre, Melbourne, VIC, Australia

**Keywords:** whole genome sequencing, *Candida glabrata*, MLST, sequence type, Australia

## Abstract

*Candida glabrata* is a pathogen with reduced susceptibility to azoles and echinocandins. Analysis by traditional multilocus sequence typing (MLST) has recognized an increasing number of sequence types (STs), which vary with geography. Little is known about STs of *C. glabrata* in Australia. Here, we utilized whole genome sequencing (WGS) to study the genetic diversity of 51 Australian *C. glabrata* isolates and sought associations between STs over two time periods (2002–2004, 2010–2017), and with susceptibility to fluconazole by principal component analysis (PCA). Antifungal susceptibility was determined using Sensititre YeastOne^TM^ Y010 methodology and WGS performed on the NextSeq 500 platform (Illumina) with *in silico* MLST STs inferred by WGS data. Single nucleotide polymorphisms (SNPs) in genes linked to echinocandin, azole and 5-fluorocytosine resistance were analyzed. Of 51 isolates, WGS identified 18 distinct STs including four novel STs (ST123, ST124, ST126, and ST127). Four STs accounted for 49% of isolates (ST3, 15.7%; ST83, 13.7%; ST7, 9.8%; ST26, 9.8%). Split-tree network analysis resolved isolates to terminal branches; many of these comprised multiple isolates from disparate geographic settings but four branches contained Australian isolates only. ST3 isolates were common in Europe, United States and now Australia, whilst ST8 and ST19, relatively frequent in the United States, were rare/absent amongst our isolates. There was no association between ST distribution (genomic similarity) and the two time periods or with fluconazole susceptibility. WGS identified mutations in the *FKS1* (S629P) and *FKS2* (S663P) genes in three, and one, echinocandin-resistant isolate(s), respectively. Both mutations confer phenotypic drug resistance. Twenty-five percent (13/51) of isolates were fluconazole-resistant (MIC ≥ 64 μg/ml) of which 9 (18%) had non wild-type MICs to voriconazole and posaconazole. Multiple SNPs were present in genes linked to azole resistance such as *CgPDR1* and *CgCDR1*, as well as several in *MSH2*; however, SNPs occurred in both azole-susceptible and azole-resistant isolates. Although no particular SNP in these genes was definitively associated with resistance, azole-resistant/non-wild type isolates had a propensity to harbor SNPs resulting in amino acid substitutions in Pdr1 beyond the first 250 amino acid positions. The presence of SNPs may be markers of STs. Our study shows the value of WGS for high-resolution sequence typing of *C. glabrata*, discovery of novel STs and potential to monitor trends in genetic diversity. WGS assessment for echinocandin resistance augments phenotypic susceptibility testing.

## Introduction

The opportunistic yeast *Candida glabrata* is the second most common cause of candidemia and invasive candidiasis (IC) in many countries (Arendrup et al., 2013; Giunea, 2014; Pfaller et al., 2014; Chapman et al., 2017). Its clinical importance as a species lies in its reduced susceptibility to azole antifungal agents; more recently, resistance to the echinocandins as well as resistance to both these drug classes (Pfaller et al., 2012; Wisplinghoff et al., 2014; Shields et al., 2015). This has prompted much investigation of the epidemiology and biological properties of *C. glabrata* infections (Vale-Silva and Sanglard, 2015).

Because the prevalence of *C. glabrata* candidiasis and drug resistance rates varies both between and within geographical region, local epidemiological data are essential to inform management (Pfaller et al., 2012; Giunea, 2014). The reasons for this variation are uncertain but likely include prior exposure to azoles, patient factors and geographic location-specific determinants. Fungus-specific factors such as genetic strain variation within species are also pertinent. Delineation of intraspecies variation is useful not only to elucidate the molecular epidemiology of *C. glabrata* infections but also to assess potential transmission routes, biological niches and population structure. Yet relatively little is known about the genomic variation between isolates from different regions or the clinical significance of such differences.

Genetic typing methods e.g., pulsed-field gel electrophoresis, microsatellite analysis and multilocus sequence typing (MLST) have been used to determine genetic relatedness of *C. glabrata* (Dodgson et al., 2003; Lin et al., 2007; Abbes et al., 2012). In particular, the use of a standardized 6-locus MLST system (Dodgson et al., 2003) has improved discrimination between isolates with good reproducibility and portability of data via internet-accessible databases. Major findings from MLST analyses highlight that despite description of a broad range of MLST sequence types (STs), *C. glabrata* appears to be highly clonal with infrequent emergence of novel STs, which may be restricted to various geographical regions (Lott et al., 2012; Hou et al., 2017; Amanloo et al., 2018). This observed clonality however, may be fluid with temporal shifts of the major *C. glabrata* subtypes documented over time in one study (Lott et al., 2010). More discriminatory methods for pathogen discrimination such as next generation sequencing (NGS) offer new insights into *C. glabrata* genetics including its molecular epidemiology and population dynamics. Global spread of previously isolated populations was inferred from genomic data in a recent study (Carrete et al., 2018). In addition, NGS has been utilized to elucidate mechanisms of drug resistance in this species from the diagnostic laboratory perspective (Singh-Babak et al., 2012; Biswas et al., 2017a).

In Australia, we observed a 1.7-fold increase in the proportion of *Candida* bloodstream infections caused by *C. glabrata* over a decade (2004–2006 vs. 2014–2015) (Chen et al., 2006; Chapman et al., 2017). Our laboratory is increasingly using whole genome sequencing (WGS) approaches, in line with international trends, in public health practice and investigations of nosocomial infections (Besser et al., 2018). Here we applied WGS to investigate the genetic diversity of Australian *C. glabrata* strains across more than a decade and sought associations between the frequency of sequence types and two time periods and with drug susceptibility to fluconazole.

## Materials and Methods

### Ethics Statement

All isolates were obtained from our culture collection spanning 10–20 years and represent previous surveillance isolates for which research ethics approval had been obtained. The present study was a laboratory-based epidemiological study. No identifiable patient data or medical records were accessed.

### Isolates and Identification

Fifty-two *C. glabrata* (sensu stricto) isolates were studied. These comprised *C. glabrata* ATCC 90030 and 51 *C. glabrata* isolates from Australia obtained through the culture collection at the Clinical Mycology Laboratory, Westmead Hospital and the Molecular Mycology Research Laboratory, Westmead Millennium Institute for Medical Research, Sydney. With the exception of two isolates recovered from the same patient 3 weeks apart, all isolates represented single patient episodes of IC. The majority (>90%) of isolates were from the jurisdictions of New South Wales and Victoria. All isolates were re-confirmed as *C. glabrata* sensu stricto by matrix-assisted laser desorption ionization-time of flight technique (MALDI-TOF MS) supplemented by ITS sequencing as required (White et al., 1990).

### Susceptibility Testing

Susceptibility to antifungal agents were determined using the Sensititre^®^YeastOne^TM^ YO10 methodology (TREK Diagnostics, Cleveland, OH, United States) according to Clinical and Laboratory Standards Institute (CLSI) methodology (Clinical and Laboratory Standards Institute [CLSI], 2017b). *Candida parapsilosis* ATCC 22019 and *Candida krusei* ATCC 6258 were the quality control strains. MIC values were interpreted according to CLSI M60 guidelines for fluconazole and the echinocandins (Clinical and Laboratory Standards Institute [CLSI], 2017a); where there are no clinical breakpoints (CBPs) (for voriconazole, posaconazole and amphotericin B), species-specific epidemiological cut-off values (ECVs) defined isolates as wild-type (WT) or non-WT (Clinical and Laboratory Standards Institute [CLSI], 2018). There are neither CBPs or ECVs for 5-fluorocytosine.

### DNA Extraction and Library Preparation for Whole Genome Sequencing

*Candida glabrata* ATCC 90030 and the 51 clinical isolates were subcultured on Sabouraud’s dextrose agar for 48 h at 35°C prior to testing to ensure purity. Genomic DNA was extracted using the Wizard^®^Genomic DNA Purification kit (Promega, Alexandria, NSW, Australia) and the concentration was quantified by Quant-iT^TM^ PicoGreen^®^dsDNA Assay Kit (Life Technologies, Mulgrave, VIC, Australia). The Nextera XT kit (Illumina, San Diego, CA, United States) was used to construct genomic libraries. Tagmentation, PCR amplification and cleanup, library normalization and pooling, and sequencing on the NextSeq 500 platform (Illumina) were carried out with 2 X 150-bp paired-end chemistry as previously described (Biswas et al., 2017b).

### Whole Genome Sequencing Data Analysis

Sequence reads were deposited in the NCBI Sequence Read Archive (SRA: project number PRJNA480138) and were mapped against the reference genome of *C. glabrata* CBS138 (GenBank Accession 4 No. GCA_0002545.2^1^).

Obtained sequence reads were mapped to each chromosome independently (using *C glabrata* CBS 138 chromosomes A to M as the reference) employing Stampy v1.0.23 (Lunter and Goodson, 2011) with pre-BWA alignment. Analysis of the mitochondria was not included. Variants were called using FreeBayes v1.1.0-dirty and filtered for read depth (minimum 20), read base quality (minimum Phred score 30), mapping quality (minimum 30) and proportion of reads supporting the variant (>0.9). All indels were excluded from the mapping-based analysis. An aligned mapped file for each chromosome was constructed for all isolates using an in-house script. All probable recombination events were identified using Gubbins (Croucher et al., 2015) and subsequently masked prior to concatenating all chromosome sequences into a single SNP alignment.

To infer the phylogenetic relationship of the Australian isolates, the best-fitting substitution model (TVM+F+ASC+R2) was selected with the Bayesian Information Criterion using ModelFinder implemented in IQ-Tree v.1.6.2 (Nguyen et al., 2015; Kalyaanamoorthy et al., 2017). A maximum likelihood tree was then reconstructed using IQ-Tree using 1000 ultrafast bootstrap replicates (Hoang et al., 2018).

To place our data into global context, all 38 publicly available *C. glabrata* Illumina short read sequence data from seven countries other than Australia were downloaded and included in the analysis (Havlesrud and Gaustad, 2017; Carrete et al., 2018; Table 2). A network approach using SplitsTree4 (Huson and Bryant, 2006) was employed to examine the relationships between our isolates and the isolates from other countries.

### Single Polynucleotide Polymorphism of Genes Associated With Antifungal Resistance

All SNPs in genes known for their role in drug resistance in *C. glabrata* (Sanguinetti et al., 2005; Berila et al., 2009; Arendrup and Perlin, 2014) were manually curated in CLC Genomics Workbench (CLC Bio version 7.0, Arrhus, Denmark) with only non-synonymous SNPs reported. Genes examined included *FKS1, FKS2, FKS3* (for echinocandin resistance) *FCY1, FCY2, CgFPS1, CgFPS2* (5-fluorocytosine resistance), *ERG9, ERG11, CgCDR1*, and Cg*PDR1* (azole resistance) and *MSH2* (for multi-drug resistance). Only non-synonymous SNPs with a minimum read depth coverage of 20, defined as high-quality (hq), were included in the analysis.

### MLST Sequence Types and Principal Component Analysis

*In silico* MLST sequence types (STs), inferred from whole genome sequence data (genome types) were obtained from assembled contigs using SPAdes v3.1.1.1 (Bankevich et al., 2012) and MLST software (Seeman, 2017, T^2^). All obtained STs were subsequently confirmed using a read based approach implemented through SRST2. Four novel *C. glabrata* MLST types (See Results) were submitted to the *C. glabrata* MLST database (Dr. Andrew Dodgson^3^ accessed September 11, 2018; and now Professor Oliver Bader, accessed November 5, 2018), and designated as ST123, ST124, ST126, and ST127. Clustering of isolates by genetic similarity according to time of isolation (2002–2004 vs. 2010–2017) and drug susceptibility (susceptible-dose-dependent (S-DD) or resistant (R) to fluconazole (Clinical and Laboratory Standards Institute [CLSI], 2017a)) was examined by Principal Component Analysis (PCA). PCA was based on a pairwise SNP distance matrix calculated under the K80 model with pairwise deletion, using the native *stats* R v3.4.1 package (R Core Team, 2013), and visualized with ggplot2 (Wickham, 2009).

## Results

Of 51 Australian clinical isolates, 49 (94%) were cultured from blood. Thirteen (25%) isolates were from the time period 2002–2004, and 38 (75%) from, 2010–2017 (Table 1).

**Table 1:** *Candida glabrata* isolates, Australia: year of isolation, body site, sequence types and *in vitro* susceptibility to five antifungal agents.

Isolate ID	Year isolation	Body site	ST	MIC μg/ml				
				
				FLU	VOR	POS	AMB	CAS
ATCC 90030	NA	Blood	ST10	8	0.5	1	1	0.06
WM_04.242	2002	Blood	ST7	32	0.5	NA	0.25	0.06
WM_03.308	2003	Blood	ST7	8	0.25	0.125	0.125	0.008
WM_03.419	2003	Blood	ST83	128^∗^	2	1	1	0.008
WM_03.449	2003	Blood	ST26	64^∗^	1	0.5	0.25	0.25
WM_03.450	2003	Blood	ST83	32	0.5	0.5	0.06	0.25
WM_03.698	2003	Blood	ST7	32	1	0.5	0.06	0.25
WM_03.707	2003	Blood	ST83	16	0.25	1	1	0.12
WM_04.113	2003	Blood	ST123	128^∗^	2	2	0.125	0.125
WM_04.387	2003	Blood	ST7	32	0.5	NA	0.25	0.25
WM_04.194	2004	Blood	ST3	4	0.25	NA	1	0.25
WM_05.155	2004	Blood	ST126	16	0.5	0.5	0.25	0.03
WM_05.111	2004	Blood	ST55	32	1	NA	0.25	1
WM_05.113	2004	Blood	ST18	64^∗^	1	NA	0.25	0.06
WM_18.26	2010	Blood	ST10	8	0.12	0.5	2	>8
WM_18.24	2012	Blood	ST16	4	0.06	0.25	0.5	8
WM_18.30	2014	Blood	ST3	16	0.5	1	1	0.25
WM_18.31	2014	Blood	ST45	1	0.06	0.12	0.5	0.03
WM_18.33	2014	Blood	ST7	128^∗^	2	>8	0.5	0.06
WM_18.36	2014	Blood	ST36	8	0.25	1	1	0.12
WM_18.39	2014	Blood	ST83	2	0.03	0.06	0.5	0.06
WM_18.40	2014	Blood	ST46	128^∗^	2	>8	1	0.06
WM_18.41	2014	Blood	ST123	8	0.25	1	0.5	0.12
WM_18.42	2014	Blood	ST123	256^∗^	8	1	0.25	0.25
WM_18.43	2014	Blood	ST83	8	0.25	1	1	0.06
WM_18.44	2014	Blood	ST3	16	0.5	2	1	0.06
WM_18.45	2014	Blood	ST127	16	0.25	1	0.5	0.06
WM_18.34	2015	Blood	ST3	16	0.5	1	1	0.06
WM_18.35	2015	Blood	ST3	4	0.12	0.25	0.5	0.03
WM_18.37	2015	Blood	ST22	16	0.25	1	0.5	0.12
WM_18.38	2015	Blood	ST6	8	0.25	1	1	0.25
WM_18.47	2015	Blood	ST46	16	0.25	0.5	1	0.25
WM_18.48	2015	Blood	ST83	32	1	2	1	0.06
WM_18.27	2015	Blood	ST26	256^∗^	8	>8	1	0.12
WM_18.29	2015	Body fluid	ST10	4	0.5	0.5	0.5	0.06
WM_18.49	2017	Blood	ST26	256^∗^	2	>8	0.5	0.12
WM_18.50	2017	Blood	ST59	8	0.25	1	1	0.06
WM_18.51	2017	Blood	ST46	128^∗^	2	>8	1	0.06
WM_18.52	2017	Body fluid	ST16	4	0.12	0.5	0.25	0.12
WM_18.53	2017	Blood	ST123	128^∗^	4	>8	1	0.12
WM_18.54	2017	Blood	ST26	0.5	0.03	0.03	1	0.25
WM_18.55	2017	Blood	ST3	8	0.25	1	1	0.25
WM_18.56	2017	Blood	ST22	32	0.5	2	0.5	0.12
WM_18.57	2017	Blood	ST16	8	0.5	2	0.5	0.12
WM_18.59	2017	Blood	ST3	16	1	2	0.5	0.12
WM_18.60	2017	Blood	ST3	8	0.5	1	0.5	0.12
WM_18.62	2017	Blood	ST55	32	2	2	1	0.06
WM_18.63	2017	Blood	ST8	2	0.12	0.25	0.5	2
WM_18.64	2017	Blood	ST8	4	0.12	0.25	0.5	2
WM_18.65	2017	Blood	ST83	64^∗^	1	2	0.5	0.12
WM_18.66	2017	Blood	ST124	256^∗^	8	2	0.125	0.25
WM_18.67	2017	Tissue	ST26	16	0.5	1	1	0.5


*ID, identification; MIC, minimum inhibitory concentration; NA, not available; ST, sequence type (refers to the MLST sequence type). ^∗^Isolates that are classed as resistant to fluconazole (Clinical and Laboratory Standards Institute [CLSI], 2017a).*

### Susceptibility Data

Table 1 summarizes the MIC values of the isolates against five antifungal agents. All clinical isolates tested had low MICs against 5-fluorocytosine (≤0.12 μg/ml) and WT MICs (<2 μg/ml) against amphotericin B. Four (7.8%) isolates (strains WM_18.26, WM_18.24, WM_18.64, and WM_18.63) were resistant to caspofungin (MIC range 2 to >8 μg/ml; Table 1), and cross resistant to micafungin and anidulafungin (results not shown) (Clinical and Laboratory Standards Institute [CLSI], 2017a). Thirteen isolates (Strains WM_03.419, WM_03.449, WM_04.113, WM_05.113, WM_18.33, WM_18.40, WM_18.27, WM_18.42, WM_18.49, WM_18.51, WM_18.53, WM_18.65, and WM_18.66) were classified as resistant to fluconazole with MICs of ≥ 64 ug/ml (Clinical and Laboratory Standards Institute [CLSI], 2017a); all also had non-WT MICs to voriconazole whilst posaconazole MICs ranged from 2 to >8 μg/ml (Table 1). However, six additional isolates (strains WM_18.44, WM_18.48, WM_18.56, WM_18.57, WM_18.59, and WM_18.62) had non-WT MICs to voriconazole and posaconazole although were susceptible-dose dependent to fluconazole, and yet 11 other isolates had non-WT MICs only to voriconazole (Table 1).

### Sequence Analysis

Overall, an average of 95% of sequencing reads were mapped to the *C. glabrata* reference genome with a median read depth coverage of 75-fold. After mapping to each reference chromosome independently, a number of recombination events were identified (range, *n* = 320–776, lowest for chromosome B and highest for chromosome I – data not shown).

### *In silico* MLST and Global Phylogeny

By WGS, there were 18 distinct STs defined based on the alleles from six genetic loci (*FKS, LEU2, NMT1, TRP1, UGP1, and URA3*) among 52 isolates (Table 1 and Supplementary Table S1), including four new STs (referred to as ST123, ST124, ST126, and ST127) not previously recognized by the *C. glabrata* MLST database (https://pubmlst.org/cglabrata/ see Supplementary Table S1 for the allele numbers of the new STs). Strain ATCC 90030 typed as ST10.

Of the known STs, the commonest ST amongst the Australian isolates was ST3 (8/51, 15.7% of isolates) followed by ST83 (7/51 and 13.7%), ST7 and ST26 (each *n* = 5, 9.8%). Collectively, these four STs were responsible for almost half (*n* = 25; 49%) of the isolates. The most common new ST was ST123 (*n* = 4 isolates). Eight (17.6%) STs (ST6, ST18, ST36, ST45, ST59, ST124, ST126, and ST127) were represented by only a single isolate (Table 1). Despite a relatively small number of isolates sequenced, the number of ST types was considerable compared to previous studies suggesting a relatively high genetic diversity within Australian *C. glabrata* isolates. Three isolates cultured from body sites other than blood were of ST10, ST16 and ST26 (Table 1). The two isolates from the same patient (WM_18.63 and WM_18.64) were both ST8.

**FIGURE 1:**
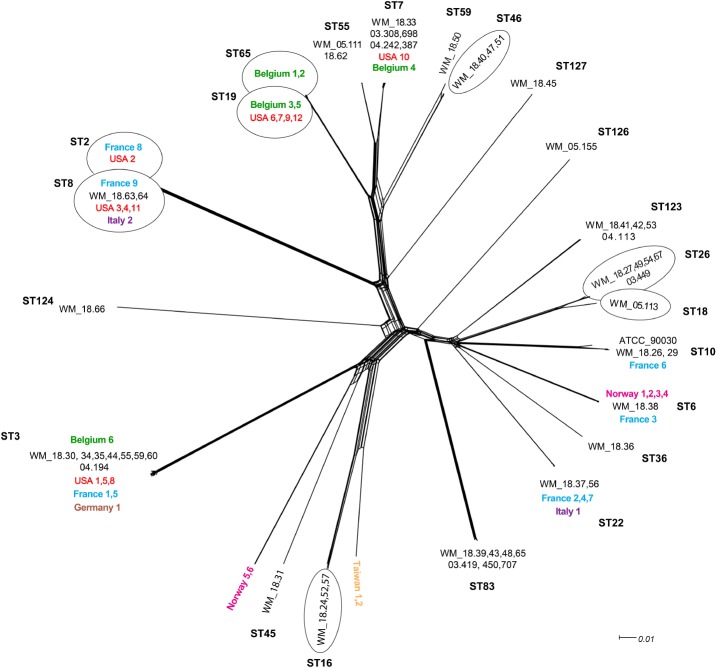
Unrooted network tree depicting the association between Australian *Candida glabrata* isolates and international isolates from seven countries based on their whole genome sequences. All clusters in the tree have been represented by different sequence types (STs) except Norway 5, Norway 6, Taiwan 1 and Taiwan 2 which have previously unassigned (new) STs. New sequence types (STs) from Australia are ST123, ST124, ST126 and ST127. Isolates representing a particular ST in branches, which contain multiple STs, are put in circles. The colors depict isolates from different countries: Black, Australia; Green, Belgium; Blue, France; Brown, Germany; Purple, Italy; Pink, Norway; Yellow, Taiwan; Red, United States. The Australian isolates have names starting with WM_ and the international isolates where named according to the country of origin, all followed by a numerical scheme. For isolates from same country in a cluster, the country name was followed by numerical identities of the isolates separated by commas. For example, in ST7 cluster, WM_18.33, 03.308,689, 04.242,387 (where 18, 03 and 04 are years of isolation followed by isolate number).

In general, the whole genome data clustered broadly within determined MLST types but with greater intra-cluster resolution. The split tree network analysis (Figure 1) resolved isolates to at least nine terminal branches containing four or more isolates; many of these comprise multiple isolates clustering together, and with isolates representing disparate geographic locations. However, four branches comprised of Australian isolates only (Figure 1). To place local isolates into a more global perspective, the STs of isolates reported from seven other countries are shown in Table 2. Certain STs were common to isolates from the regions studied herein e.g., ST3 (Belgium, France, Germany, United States, Australia), whilst others were either more restricted, or were more prevalent to one or two countries e.g., ST6 in Norway and France, ST8 in the United States and continental Europe. The two isolates from Taiwan showed new STs. Of note, isolates of ST19 were absent from Australia (vs. 4/12 US isolates; Table 2).

**Table 2:** *Candida glabrata* isolates from countries other than Australia with known multi-locus sequence types as a comparison with sequence types of Australian isolates.

Isolate ID^∗^	SRA Run ID	Country	Body site	Sequence Type
Belgium1	SRR5239784	Belgium	Mouth	ST65
Belgium2	SRR5239783	Belgium	Stool	ST65
Belgium3	SRR5239781	Belgium	Stool	ST19
Belgium4	SRR5239776	Belgium	Stool	ST7
Belgium5	SRR5239782	Belgium	Mouth	ST19
Belgium6	SRR5239756	Belgium	Mouth	ST3
France1	SRR5239754	France	Blood	ST3
France2	SRR5239767	France	Blood	ST22
France3	SRR5239764	France	Blood	ST6
France4	SRR5239768	France	Blood	ST22
France5	SRR5239755	France	Blood	ST3
France6	SRR5239765	France	Blood	ST10
France7	SRR5239766	France	Stool	ST22
France8	SRR5239773	France	Blood	ST2
France9	SRR5239758	France	Blood	ST8
Germany1	SRR5239759	France	Stool	ST3
Italy1	SRR5239769	Italy	Blood	ST22
Italy2	SRR5239770	Italy	Blood	ST8
Norway1	SRR2982714	Norway	Blood	ST6
Norway2	SRR2982715	Norway	Blood	ST6
Norway3	SRR2982716	Norway	Blood	ST6
Norway4	SRR2982717	Norway	Blood	ST6
Norway5	SRR2982718	Norway	Blood	NEW ST
Norway6	SRR2982719	Norway	Blood	NEW ST
Taiwan1	SRR5239763	Taiwan	Mouth	NEW ST
Taiwan2	SRR5239762	Taiwan	Mouth	NEW ST
USA1	SRR5239757	United States	Blood	ST3
USA2	SRR5239772	United States	Blood	ST2
USA3	SRR5239774	United States	Blood	ST8
USA4	SRR5239753	United States	Blood	ST8
USA5	SRR5239761	United States	Blood	ST3
USA6	SRR5239779	United States	Blood	ST19
USA7	SRR5239777	United States	Blood	ST19
USA8	SRR5239760	United States	Blood	ST3
USA9	SRR5239778	United States	Blood	ST19
USA10	SRR5239775	United States	Blood	ST7
USA11	SRR5239771	United States	Blood	ST8
USA12	SRR5239780	United States	Blood	ST19


*ID, identification; ST, sequence type; USA, United States of America. ^∗^NEW ST: Sequence type reported as novel (Carrete et al., 2018).*

### Genomic Similarity According to Period of Isolation and Susceptibility to Fluconazole

**FIGURE 2:**
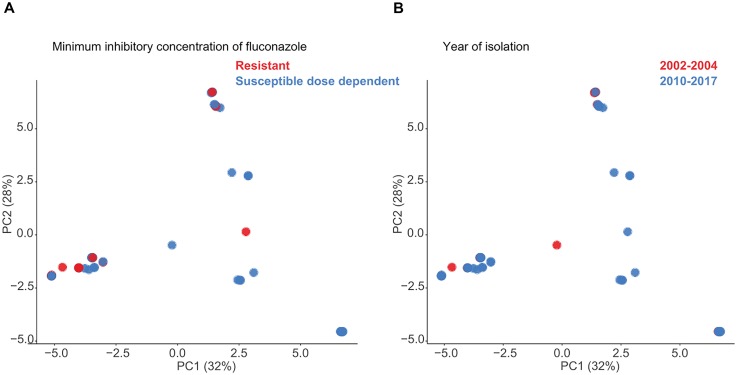
Principle component analysis (PCA) of *Candida glabrata* genomic SNP distances following masking of recombination and **(A)** phenotypic fluconazole susceptibility or **(B)** period of isolation.

On analysis of the sequenced genomes by PCA, there was no temporal association between the two periods of isolation (2002–2004 and 2010–2017) and genomic similarity (as represented by ST distribution), or between genomic similarity and fluconazole susceptibility as measured by MICs (fluconazole S-DD: *n* = 39 isolates vs. fluconazole-resistant: *n* = 12) (Figures 2A,B).

**FIGURE 3:**
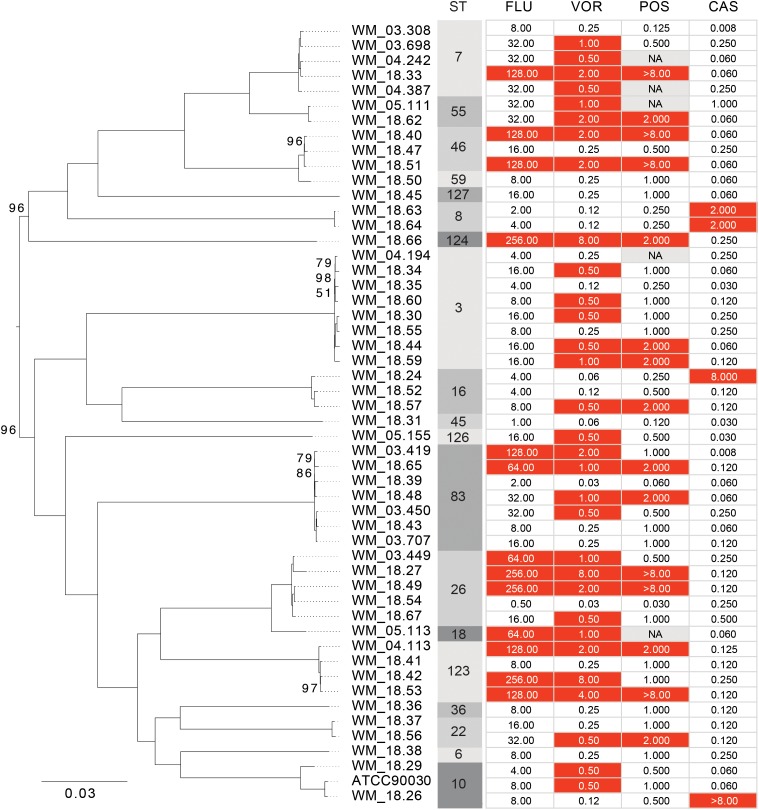
Maximum likelihood phylogeny of Australian *Candida glabrata* isolates. Bootstrap support values of less than 100 are indicated at the corresponding nodes. Sequence types (STs) and minimum inhibitory concentrations (MICs) for fluconazole (FLU), voriconazole (VOR), posaconazole (POS) and caspofungin (CAS) of each isolate is indicated to the right of the tree. Isolates with MIC values that are classed as “resistant” or as “non-wild type” are colored in red.

The phylogenetic relationship of the 51 Australian isolates was also reconstructed with high bootstrap support (Figure 3). There was no association between drug susceptibility to fluconazole, voriconazole, posaconazole or caspofungin (results were similar for anidulafungin and micafungin) or resistance, and phylogenetic clustering. Rather the analysis illustrated that resistance or non-WT MICs emerged at several time points along the phylogeny.

### Analysis for SNPS Related to Drug Resistance and Their Relationship to STs

Of two isolates with caspofungin MICs of ≥ 8 μg/ml, one (strain WM_18.24; ST16) contained the *FKS1* mutation leading to the amino acid substitution Ser629Pro, whilst strain WM_18.26 (ST10) harbored the *FKS2* mutation Ser663Pro. There were no other SNPs in any of *FKS1, FKS2* or *FKS3* in both strains. Isolates WM_18.63, and WM-18.64 (both ST8) recovered from the same patient harbored the *FKS1* mutation Ser629Pro as well as a *FKS2* mutation Glu784Gly. The Glu784Gly mutation was not present in any echinocandin susceptible isolates. Other SNPs were present in *FKS1* (Gly14Ser) and *FKS2* (Thr926Pro) but only in isolates of ST3. Several SNPs in *FKS3* (Supplementary Table S2) were present in both echinocandin-susceptible and echinocandin-resistant isolates.

The presence of SNPs in genes linked to 5-fluorocytosine resistance e.g., *CgFPS1, CgFPS2* and *CgFCY1* and *CgFCY2* broadly, varied with ST with no SNPs observed in isolates of ST6, ST22, ST55, ST59 and ST123 (Supplementary Table S2). Fluconazole-resistant isolates (Table 1) in general harbored mutations in *CgPDR1* and to a lesser extent in *CgCDR1*, but overall, SNPs in these genes and in other efflux pump genes, *CgFLR1* and *CgSNQ2* (data not shown) were also present in azole-susceptible/WT isolates (Supplementary Table S2). However, although no particular SNP was definitively linked to the resistance phenotype, 5/13 fluconazole-resistant isolates harbored Pdr1 amino acid substitutions in the region beyond the first 200–250 amino acids with no fluconazole-susceptible isolate containing such changes. In addition, 2/6 isolates with non-WT MICs to voriconazole and posaconazole (strains WM_18.48 and WM_ 18.62; fluconazole MIC both 32 μg/ml) also harbored mutations resulting in amino acid substitutions outside this region. Conversely, substitutions within the first 250 amino acid positions in Pdr1 were common to both azole resistant and susceptible isolates (Supplementary Table S2). Eight fluconazole resistant isolates however, did not demonstrate a *PDR1* mutation. Isolates exhibiting pan-azole resistance, or which had non-WT MICs also had mutation in *CgCDR1* His58Tyr (6/10 isolates), but the last was also present in azole-susceptible isolates.

SNPs occurred in isolates of diverse ST. There were no SNPs in *ERG11* and the few SNPs observed in *ERG9* were predominantly in isolates of ST3 and ST26.

SNPs in the *MSH2* gene were observed in 19 of 51 (37%) isolates with three main locations of mutations – Val239Leu (9 isolates), Glu456Asp (7 isolates) and Leu269Phe (3 isolates), with two isolates (strains WM_18.63 and WM_18.64) having two mutations at Val239Leu and Ala942Thr (Supplementary Table S2). The same *MSH2* mutations were found in azole susceptible as well as azole-resistant/non-WT isolates. Overall, SNPs were identified in isolates of diverse STs including in isolates of the new STs, ST123 and ST127 (Supplementary Table S2). Whilst the mutation Glu456Asp was found in isolates of 5 different STs and that of Val239Leu in 4 different STs, the Leu269Phe mutation was found only in isolates of ST16. The combination of Val239Leu and Ala942Thr were only identified in ST8 isolates, both of which were pan-echinocandin resistant.

## Discussion

Understanding the genomic diversity of *C. glabrata* and its antifungal susceptibility patterns is key to optimal management of infections caused by this problematic pathogen. The few studies that have examined the genetic variation of large culture collections have employed traditional MLST and indicate a predominantly clonal population structure with infrequent recombination (Dodgson et al., 2005). Prevalence of circulating STs also showed geographical bias (Dodgson et al., 2003; Hou et al., 2017; Amanloo et al., 2018). Hence, genetic variation amongst isolates from one region cannot be generalized to another. Here, we determined for the first time using a WGS approach, the relative frequency of endemic STs among 51 Australian *C. glabrata* isolates from two time periods, and verified the applicability of WGS to determine STs, STs by WGS clustered isolates within similar “ST” groupings as *in silico* MLST with good intra-cluster resolution (Figure 1).

Sequence typing demonstrated relatively “large” genetic diversity amongst Australian *C. glabrata* isolates, with just under half of the isolates represented by only four STs. The remaining STs, not only represented Australian specific STs (Figures 1, 3) but suggested an overall diversity within this pathogen that is greater than previously appreciated. In a nationwide Chinese study (Hou et al., 2017) of 411 isolates, a “new” ST sequence type was encountered approximately every 11 isolates compared to our study, which observed a “new” ST every 5 isolates. These, and our data emphasize the regional differences, with 75.9% of Chinese isolates comprised of ST7 and ST3 compared to only 25.5% of Australian isolates. Another recent report noted a predominance of ST3 and ST7 (70% isolates) in Korea (Byun et al., 2018) whilst in Iran, three STs (ST59, ST74, and ST7) accounted for 50% of isolates further supporting the notion of low intraspecies diversity within *C. glabrata* (Amanloo et al., 2018). Isolates belonging to ST3 have been reported with relative high frequency from Europe and Asia and now, from Australia (Dodgson et al., 2003; this study). The presence of strains with the same ST on different continents demonstrates that clones may have arisen from the same ancestor and disseminated globally followed by local adaptation.

Whilst ST5 isolates were reportedly common in Europe (Dodgson et al., 2003), this ST was not found amongst our Australian isolates nor amongst those from a more recent study of European and US isolates (Tables 1, 2). Conversely, isolates of ST7 appear uncommon in Europe and the United States but are more prevalent in Japan, Korea and China (Dodgson et al., 2003; Hou et al., 2017; Byun et al., 2018; Carrete et al., 2018). Strains of ST8, ST18, and ST19 were the commonest types in the United States (Dodgson et al., 2003) whereas we identified only one ST18 isolate, no ST19 isolates and two ST8 isolates (from the same patient) in Australia. Broadly, there are more common STs between Australian and Asian isolates than between Australian and US/European isolates likely reflecting the geographical proximity between Asia and Australia. Further studies involving a larger number of *C. glabrata* isolates to test this hypothesis would be of interest. The observed geographic variation among STs highlights the importance of acquiring local data.

MLST analysis of US *C. glabrata* isolates collected over three time periods between 1992–2009 revealed a relatively small number of STs with little genetic differentiation (Lott et al., 2010). In the present study, the results indicate that there is no evidence of genomic similarity or ST distribution among the sequences isolated in the two timeframes studied (Figure 2A), suggesting that there have been no marked shifts in the present STs. We further found no association between WGS STs and susceptibility or /resistance to fluconazole (Figure 2B), consistent with that reported by traditional MLST studies (Dodgson et al., 2003; Amanloo et al., 2018). The phylogenetic tree (Figure 3) suggests that both azole and echinocandin resistance may arise at multiple time points, independent of strain clustering. The small numbers of isolates are acknowledged as a study limitation.

Strain typing is essential for epidemiological investigation. MLST has the advantage of providing easily comparable results via internet-accessible databases (Dodgson et al., 2003), but does not adequately capture the breadth of genetic diversity and is not readily available in diagnostic laboratories. The present study has illustrated the utility of WGS to delineate genome variability in *C. glabrata* and importantly offers both superior discriminatory power and convenience. The costs of the two techniques are nearly identical – approximately AUD 50/sample for MLST and AUD 80/sample for WGS. With decreasing footprint and technological advances, however, cost reduction for WGS is anticipated.

The echinocandin resistance rate of 7.8% (4/51 isolates) in the present study is influenced by sampling bias, being <2% across Australia (Chapman et al., 2017), lower than that in the United States (Alexander et al., 2013; Shields et al., 2015). The *FKS1* mutation Ser629Pro (in one isolate) and the mutation *FKS2* Ser663Pro (three isolates) identified are among the most common in *C. glabrata* strains with high-level resistance phenotypes (Garcia-Effron et al., 2009; Arendrup and Perlin, 2014). None of the isolates harbored other well-known mutations that confer echinocandin resistance e.g., R665G, R636S, and F659Y (Zimbeck et al., 2010; Shields et al., 2015). Conversely, the role of *FKS2* Glu748Gly in isolates WM_18.63 and WM_18.64 in echinocandin resistance remains uncertain as this SNP has not been previously described. Interestingly, the SNP was absent in genomes of all ST8 isolates from other countries in that cluster (Italy2, France9, USA3, USA4, and USA11).

Approximately 25% of *C. glabrata* isolates in our study were fluconazole-resistant, comparable to that in the United States (20–30%) (Castanheira et al., 2014). Genome-wide sequencing revealed mutations in several multidrug resistance transporter genes (Supplementary Table S2) (e.g., *CgPDR1* and *CgCDR1*) that are associated with resistance through activation of drug efflux pumps (Ferrari et al., 2009; Rodrigues et al., 2014). Although SNPs in these genes were found in both azole-resistant/non wild-type as well as azole-susceptible/wild-type isolates, as previously reported, the alterations were located within the first ∼250 amino acids of Pdr1 in both susceptible and resistant isolates. It is noteworthy that 5/13 fluconazole-resistant isolates had other amino acid substitutions located outside this region of Pdr1 (after the first 250 amino acid positions) (Supplementary Table S2) (Ferrari et al., 2009; Tsai et al., 2010; this study). The reasons for fluconazole resistance in the remaining 8/13 isolates lacking Pdr1 mutations are uncertain. One possibility is that these isolates are petite mutants due to the loss of functional mitochondria, common in *C. glabrata* upon fluconazole exposure (Brun et al., 2004; Ferrari et al., 2009). However, effects of specific SNPs in these genes and the potential of petite mutants leading to fluconazole resistance phenotype needs to be confirmed by functional and gene expression analyses, which was beyond the scope of the present study. Through gene deletion studies, it has been recently shown that when functionally active, all of *CDR1, PDR1* and *SNQ2* contribute to high level resistance to azoles (Whaley et al., 2018). The absence of *ERG11* mutations predominant in other *Candida* species, such as azole-resistant *C. albicans* is also important (Morio et al., 2010).

Mutations in the DNA mismatch repair gene *MSH2* are reported to be a genetic driver of multi-drug resistance and about 55% of *C. glabrata* isolates are expected to contain *MSH2* gene mutations. Whilst over 35% of our isolates harbored a mutation in *MSH2*, these were present in both azole-susceptible and azole-resistant (or non-WT) isolates. Hence it is possible that *MSH2* mutations are more a marker of “ST” of *C. glabrata* rather than an indicator of drug resistance, having been linked to isolates of ST16 (Healey et al., 2016; Delliére et al., 2016); similarly, in our study, the mutation Leu269Phe was only present in isolates of ST16 (Supplementary Table S2). However, the remaining *MSH2* mutations were found in several STs including two of the novel STs identified herein. Of note, the mutation combination of Val239Leu and Ala942Thr were only identified in ST8.

Limitations of the present study include the relatively small numbers of isolates analyzed, which may have precluded the identification of associations between ST and period of isolation. In addition, the majority of isolates were from blood. However, Lott et al. demonstrated that bloodstream isolates of *C. glabrata* were genetically indistinguishable from those colonizing the host (Lott et al., 2012). By using genome-wide information in 33 strains, Carrete et al. inferred the population structure of *C. glabrata* where strains were clustered into highly divergent clades but with the structure suggesting recent global spread of previously isolated populations (Carrete et al., 2018). WGS with its superior discrimination is well placed to provide additional clues to evolutionary traits in this species.

## Conclusion

In conclusion, we have shown the value of a WGS approach for high resolution sequence typing, discovery of novel STs of *C. glabrata*, and the potential to monitor trends in genetic diversity. We envisage useful contribution of our data including that of four novel STs to the global sequence repository. Our results suggest that azole, as well as echinocandin, resistance may arise at multiple time points independent of strain clustering or of STs. WGS assessment for echinocandin resistance has good potential to augment phenotypic susceptibility testing methods. Further study by WGS of *C. glabrata* STs and their evolution over time is warranted.

## Author Contributions

SC, VS, WM, CB, SV, VM, and CH conceived and designed the research. CB, VM, QW, EM, CH, SV, SC, VS, and WM performed the experimental work and analysis of genomic data. CB, CH, WM, SV, SC, SK, KK, DM, MS, IA, CM, and TS provided isolates and analyzed the data. SC, CB, VM, and SV wrote the manuscript. All authors read, provided scientific critique, edited, and approved the manuscript.

## Conflict of Interest Statement

SV, MS, DM, CM, KK, TS, SK, CH, and IA are part of antifungal advisory boards and untied grants from MSD Australia and Gilead Sciences Inc. The remaining authors declare that the research was conducted in the absence of any commercial or financial relationships that could be construed as a potential conflict of interest.

## Footnotes

1 http://www.candidagenome.org

2 https://github.com/tseemann/mlst

3 https://pubmlst.org/cglabrata/
