# Corrigendum: Whole Genome Sequencing of Australian *Candida glabrata* Isolates Reveals Genetic Diversity and Novel Sequence Types

**DOI:** 10.3389/fmicb.2019.02218

**Published:** 2019-09-27

**Authors:** Chayanika Biswas, Vanessa R. Marcelino, Sebastiaan Van Hal, Catriona Halliday, Elena Martinez, Qinning Wang, Sarah Kidd, Karina Kennedy, Deborah Marriott, C. Orla Morrissey, Ian Arthur, Kerry Weeks, Monica A. Slavin, Tania C. Sorrell, Vitali Sintchenko, Wieland Meyer, Sharon C.-A. Chen

**Affiliations:** ^1^Centre for Infectious Diseases and Microbiology-Public Health, Westmead Hospital, Sydney, NSW, Australia; ^2^Westmead Clinical School, Faculty of Medicine and Health, The University of Sydney, Sydney, NSW, Australia; ^3^Centre for Infectious Diseases and Microbiology, Westmead Institute for Medical Research, Westmead, NSW, Australia; ^4^Marie Bashir Institute for Emerging Infectious Diseases and Biosecurity, The University of Sydney, Sydney, NSW, Australia; ^5^Department of Infectious Diseases and Microbiology, New South Wales Health Pathology, Royal Prince Alfred Hospital, Faculty of Medicine and Health, The University of Sydney, Sydney, NSW, Australia; ^6^Centre for Infectious Diseases and Microbiology Laboratory Services, ICPMR, New South Wales Health Pathology, Westmead Hospital, Sydney, NSW, Australia; ^7^National Mycology Reference Centre, SA Pathology, Adelaide, SA, Australia; ^8^Department of Microbiology and Infectious Diseases, Canberra Hospital & Health Services, Australian National University Medical School, Canberra, ACT, Australia; ^9^Department of Microbiology and Infectious Diseases, St Vincent's Hospital, Sydney, NSW, Australia; ^10^Department of Infectious Diseases, Alfred Health and Monash University, Melbourne, VIC, Australia; ^11^Department of Microbiology, PathWest Laboratory Medicine, Queen Elizabeth II Medical Centre, Perth, WA, Australia; ^12^Department of Microbiology and Infectious Diseases, Royal North Shore Hospital, Sydney, NSW, Australia; ^13^National Centre for Infections in Cancer, Peter MacCallum Cancer Centre, Melbourne, VIC, Australia

**Keywords:** whole genome sequencing, *Candida glabrata*, MLST, sequence type, Australia

In the original article, there were a small number of transcription errors in Figure 1 as published.

There are:

The label “Norway 6” (at position 6 o'clock) was incorrectly “positioned” with isolate WM_18.31 (ST45) and should have been positioned with “Norway 5.” This is now corrected.

Isolates Taiwan 1 and Taiwan 2 should be positioned together. In addition, the isolate WM_18.57 was inadvertently omitted from the ST16 “bubble.”

Isolate WM_18.51 should have been placed in the group under ST46 instead of ST59. The corrected Figure 1 appears below.

**Figure 1:**
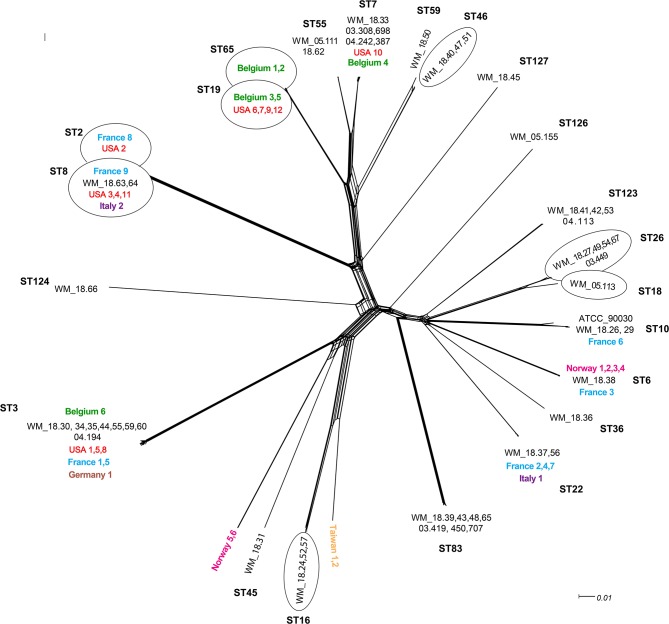
Unrooted network tree depicting the association between Australian *Candida glabrata* isolates and international isolates from seven countries based on their whole genome sequences. All clusters in the tree have been represented by different sequence types (STs) except Norway 5, Norway 6, Taiwan 1 and Taiwan 2 which have previously unassigned (new) STs. New sequence types (STs) from Australia are ST123, ST124, ST126 and ST127. Isolates representing a particular ST in branches, which contain multiple STs, are put in circles. The colors depict isolates from different countries: Black, Australia; Green, Belgium; Blue, France; Brown, Germany; Purple, Italy; Pink, Norway; Yellow, Taiwan; Red, United States. The Australian isolates have names starting with WM_ and the international isolates where named according to the country of origin, all followed by a numerical scheme. For isolates from same country in a cluster, the country name was followed by numerical identities of the isolates separated by commas. For example, in ST7 cluster, WM_18.33, 03.308,689, 04.242,387 (where 18, 03 and 04 are years of isolation followed by isolate number).

The authors apologize for these errors and state that they do not change the scientific findings of the article in any way. The original article has been updated.

