# Supplementary material for: Whole Genome Sequencing of Australian Candida glabrata Isolates Reveals Genetic Diversity and Novel Sequence Types
Source: Front Microbiol. 2018 Dec 3;9:2946. doi: 10.3389/fmicb.2018.02946 (PMC6287553; doi:10.3389/fmicb.2018.02946)
Supplement: Supplementary file 1 [file Table_1.docx]

**Supplementary Table S1:** Multi-locus sequence type (MLST) profiles for Australian *Candida glabrata* isolates with novel sequence types.

| Isolate number | Source | Sequence type  (ST) | Allele  *FKS* | Allele  *LEU2* | Allele  *NMT1* | Allele  *TRP1* | Allele  *UGP1* | Allele  *URA3* |
| --- | --- | --- | --- | --- | --- | --- | --- | --- |
| WM18.53 | Blood | ST123 | 7 | 16 | 34* | 13 | 1 | 8 |
| WM18.41 | Blood | ST123 | 7 | 16 | 34* | 13 | 1 | 8 |
| WM18.42 | Blood | ST123 | 7 | 16 | 34* | 13 | 1 | 8 |
| WM04.113 | Blood | ST123 | 7 | 16 | 34* | 13 | 1 | 8 |
| WM18.66 | Blood | ST124 | 8* | 3* | 9* | 13 | 11* | 9* |
| WM05.155 | Blood | ST126 | 13 | 2 | 9* | 4 | 13 | 9 |
| WM18.45 | Blood | ST127 | 14 | 12 | 14* | 16* | 3 | 10 |

*Indicates a novel allele which contains at least ≥1 mismatch from the closest matching known allele

All ST types within the table represent unique combinations of existing alleles and/or novel alleles (https://pubmlst.org/cglabrata/).
